# Vitreous hemorrhage in retinal vein occlusion without visible traction from the posterior vitreous membrane: An optical coherence tomography angiography case report study

**DOI:** 10.1016/j.heliyon.2024.e26019

**Published:** 2024-02-10

**Authors:** Yuki Akiyama, Yuki Muraoka, Takahiro Kogo, Naomi Nishigori, Masaharu Ishikura, Kenji Ishihara, Tomoaki Murakami, Sotaro Ooto, Akitaka Tsujikawa

**Affiliations:** Department of Ophthalmology and Visual Sciences, Kyoto University Graduate School of Medicine, Kyoto, Japan

## Abstract

**Background:**

We report an unusual case of retinal vein occlusion (RVO) associated with vitreous hemorrhage (VH) without visible traction from the posterior vitreous membrane (PVM) at the bleeding point, challenging our current understanding of VH pathophysiology.

**Case presentation:**

A 52-year-old man presented with VH in the right eye. A detailed examination using optical coherence tomography angiography (OCTA) and ultra-widefield fluorescein angiography revealed branch RVO with non-perfused areas (NPAs) extending peripherally and neovascularization elsewhere (NVE). OCTA showed NVE infiltrating the vitreous cavity, leading to substantial bleeding without visible PVM traction at the bleeding point. The NVE was successfully removed following vitrectomy, and visual acuity improved from 20/20 to 20/13 preoperatively, along with a postoperative improvement in floaters.

**Conclusions:**

This unique case of RVO suggests the possibility of VH occurring independent of PVM contractions at the bleeding point, challenging the traditional understanding of VH. This finding underscores the potential role of OCTA in diagnosing and managing retinal vascular diseases, underscoring the need for further investigations into the underlying mechanisms, with potential implications for personalized therapeutic strategies.

## Highlights

• Retinal vein occlusion often results in retinal non-perfused areas.
• Ultra-widefield fluorescein angiography could detect retinal non-perfused areas.
• The bleeding point lacked visible posterior vitreous membrane traction.
• Vitrectomy removed the neovascularization and improved visual acuity.

## 1 Introduction

Retinal circulatory disorders, such as diabetic retinopathy and retinal vein occlusion (RVO), often lead to non-perfused areas (NPAs) within the retina [1,2]. These NPAs can stimulate the secretion of growth factors, such as vascular endothelial growth factor, owing to the associated hypoxia and ischemia [1]. Retinal neovascularization elsewhere (NVE) may occur [3] in extensive NPAs, which do not comprise posterior vitreous detachment, by utilizing the posterior vitreous membrane (PVM) as a scaffold for growth [4].

In cases of branch RVO (BRVO), a substantial proportion of patients develop NVE within 3 years [3], frequently resulting in vitreous hemorrhage (VH) [5]. Traditionally, VH is considered to originate from the inherent fragility of the NVE, combined with the mechanical traction exerted by the PVM.

However, the conventional understanding of VH pathogenesis remains unclear, given the inherent challenges the hemorrhage poses in visualizing the vitreoretinal interface. In the current report, we present a case of BRVO, in which a minimal VH enabled the effective visualization of the PVM and NVE using optical coherence tomography angiography (OCTA). Interestingly, the observed VH occurred without clear evidence of PVM traction. The present case suggests that physical traction is not always a mandatory prerequisite for VH development, as previously presumed, and underscores the need for continued investigations into the underlying mechanism.

## 2 Case presentation

**Fig. 1:**
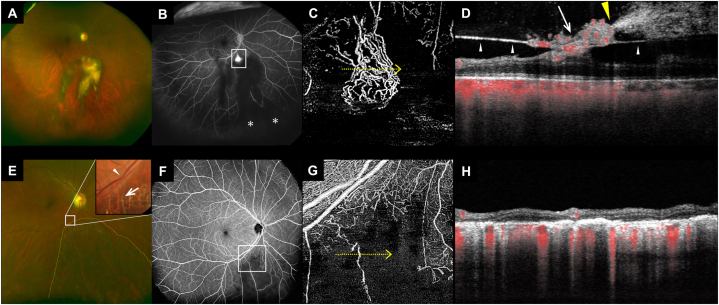
Preoperative and postoperative fundus images of a patient with branch retinal vein occlusion accompanied by retinal neovascularization elsewhere (NVE) and vitreous hemorrhage (VH) A–D: Preoperative images. A: Wide-field scanning laser ophthalmoscopy (SLO) image depicting organized VH inferior to the optic disc. B: Ultra-widefield fluorescein angiography image depicting the NVE (square) inferior to the optic disc. The retinal nonperfusion area (*) is visible in the lower peripheral region, despite being obscured by the VH. C: Optical coherence tomography angiography (OCTA) vitreoretinal interface slab image depicting distinct NVE. D: OCT along a yellow arrow (in panel C) depicting the posterior vitreous membrane (PVM, white arrowheads) and VH originating from a specific point (yellow arrowhead) within the NVE (white arrow). OCTA does not indicate visible traction from PVM at this bleeding point. E–H: Postoperative fundus images obtained after vitrectomy. E: Wide-field SLO image depicting the retinal area beneath the optic disc, highlighting the laser photocoagulation spots (yellow dotted line). An inset color fundus photograph in the top right corner offers an enlarged view of the venous occlusion site (white arrowhead) and ensuing whitened vessels (white arrow). F: Wide-field OCTA image depicting a large avascular area indicates a successful NVE removal. G: Postoperative OCTA superficial retinal slab image. H: OCT along a yellow arrow (in panel G). (For interpretation of the references to color in this figure legend, the reader is referred to the Web version of this article.)

A 52-year-old man with well-controlled hypertension and no prior ophthalmic history presented with an increased number of floaters in the right eye. He did not have a history of diabetes mellitus. Upon examination, we identified an organized VH (Fig. 1). Utilizing color fundus photography, OCTA, and ultra-widefield fluorescein angiography, we detected the affected arteriovenous crossing site: an NVE below the optic disc and peripheral NPAs in the quadrant comprising the NVE, indicative of an old BRVO. No other chorioretinal diseases that could be associated with VH, including retinal tears or retinal arterial macroaneurysms, were observed. OCT B-scan found no evidence of posterior vitreous detachment. However, OCTA indicated an NVE extension through the PVM into the vitreous cavity, with the VH apparently originating from a specific point within the NVE. Of note, upon a comprehensive review of all OCTA slices, no visible PVM traction was detected at the bleeding site.

At the initial visit, the best corrected visual acuity of the right eye was 20/20. In the 5 weeks leading to the intervention, there was no exacerbation of vitreous hemorrhage-related symptoms, such as increased floaters or decreased visual acuity. After retinal photocoagulation and pars plana vitrectomy for the NVE and NPAs, visual acuity improved to 20/13, and floaters improved. Postoperatively, enhanced visibility allowed for precise localization of the affected arteriovenous crossing site and its whitened vessels (Fig. 1E), which substantiated the diagnosis of BRVO. The NVE was removed successfully, and NPAs were outlined distinctly using ultra-widefield OCTA.

## 3 Discussion

VH frequently accompanies diseases affecting retinal circulation, highlighting a crucial interaction between neovascularization and the vitreous environment. The present report discusses an unusual case of RVO that provides insights into the diverse pathophysiology of VH and the involvement of NVE.

Typically, VH occurs when new fragile vessels are damaged by vitreous traction [4,5]. This commonly leads to bleeding throughout the vitreous cavity, thus complicating fundus imaging. The present case offers a different perspective, wherein the attachment point of PVM is positioned closer to the retina than to the point of hemorrhage from the NVE. Typically, VH associated with NVE occurs when the PVM is anchored to the retina, generating a pulling force at the attachment site. This traction often leads to stress at the site, potentially causing damage to new vessels and subsequent VH. In our case, however, hemorrhage was observed slightly farther (i.e., anteriorly) from the PVM attachment point, suggesting a hemorrhagic pattern different from conventional, traction-related bleeding. The potential significance of this finding is that it challenges the prevailing belief that PVM traction is a necessary precondition for VH.

Vaz-Pereira et al. [6] have categorized NVE in proliferative diabetic retinopathy into flat, forward, and tabletop types based on the OCT findings. The current case may represent a forward-type NVE invading the vitreous. The forward-type NVE, which is considered more fragile and capable of causing VH, aligns with the observations reported by Cui et al. [7] However, determining the type of mechanical stress inducing NVE failure and bleeding based solely on images remains challenging.

Typically, VH management entails vitrectomy, which includes inducing artificial posterior vitreous detachment, and NVE removal. However, an alternative strategy might have been more appropriate in the current case because of its inherent invasiveness and the absence of PVM traction at the presumed bleeding point. Specifically, anti-vascular endothelial growth factor therapy may have achieved substantial NVE regression.

The current case suggests that NVE can cause VH without the influence of PVM contraction and underscores the potential of OCTA for evaluating NVE and vitreoretinal interface. However, it must be acknowledged that assessing all mechanical stresses solely through OCTA remains challenging, and image-based analysis without supporting clinical or histopathological data inherently holds limitations. Furthermore, it highlights the need for additional research to comprehensively clarify underlying mechanisms, assess the prevalence of similar cases among patients with retinal vascular diseases, and evaluate the potential role of OCTA in diagnosis, monitoring, and treatment.

## 4 Conclusion

In summary, this unusual case of RVO can contribute to understanding diverse pathophysiological mechanisms underlying VH. It underscores the need for further exploring such unusual presentations and suggests that a more detailed understanding could facilitate the development of personalized therapeutic strategies, thereby improving patient care.

## Ethics statements

PATIENT CONSENT: The patient provided written consent to publish this case report.

## Data availability statement

The datasets used and/or analyzed during the current study are available from the corresponding author upon reasonable request.

## CRediT authorship contribution statement

**Yuki Akiyama:** Conceptualization, Data curation, Visualization, Writing – original draft. **Yuki Muraoka:** Conceptualization, Project administration, Writing – review & editing. **Takahiro Kogo:** Writing – review & editing. **Naomi Nishigori:** Writing – review & editing. **Masaharu Ishikura:** Writing – review & editing. **Kenji Ishihara:** Writing – review & editing. **Tomoaki Murakami:** Writing – review & editing. **Sotaro Ooto:** Writing – review & editing. **Akitaka Tsujikawa:** Supervision, Writing – review & editing.

## Declaration of competing interest

The authors declare the following financial interests/personal relationships which may be considered as potential competing interests:Yuki Muraoka has received grants and financial support from 10.13039/100015731Bayer Yakuhin, 10.13039/100008792Novartis Pharma, Canon, 10.13039/501100004286Santen Pharmaceutical, Senju Pharmaceutical, 10.13039/100007816Alcon Japan, and 10.13039/100006116AMO Japan. Tomoaki Murakami has received grant and financial support from 10.13039/100015731Bayer Yakuhin, Kowa Pharmaceutical, 10.13039/100007816Alcon Pharma, 10.13039/100008792Novartis Pharma, 10.13039/100006116AMO Japan, 10.13039/501100004286Santen Pharmaceutical, 10.13039/100007816Alcon Japan, and Senju Pharmaceutical. Sotaro Ooto has received grant and financial support from 10.13039/100015731Bayer Yakuhin, Kowa Pharmaceutical, 10.13039/100007816Alcon Pharma, Janssen Pharmaceutical, 10.13039/100008792Novartis Pharma, 10.13039/100006116AMO Japan, 10.13039/501100004286Santen Pharmaceutical, 10.13039/100007816Alcon Japan, Senju Pharmaceutical, and Japan 10.13039/501100014065Focus. Akitaka Tsujikawa has received grants and financial support from Canon, Findex, 10.13039/501100004286Santen Pharmaceutical, Kowa Pharmaceutical, 10.13039/100004319Pfizer, 10.13039/100006116AMO Japan, Senju Pharmaceutical, 10.13039/501100004419Wakamoto Pharmaceutical, 10.13039/100007816Alcon Japan, 10.13039/100007816Alcon Pharma, 10.13039/501100007132Otsuka Pharmaceutical, Tomey Corporation, 10.13039/100009954Taiho Pharma, HOYA, 10.13039/100015731Bayer Yakuhin, 10.13039/100008792Novartis Pharma, 10.13039/100010795Chugai Pharmaceutical, 10.13039/501100004948Astellas, 10.13039/501100003769Eisai, Daiich-Sankyo, Janssen Pharmaceutical, Kyoto Drug Discovery & Development, 10.13039/100007819Allergan Japan, MSD, Ellex, Sanwa Kagaku Kenkyusho, Nitten Pharmaceutical, and 10.13039/100006483AbbVie 10.13039/100013093GK. YA, KT, NN, MI and KI have no financial disclosures.
